# Tracking working status of HIV/AIDS-trained service providers by means of a training information monitoring system in Ethiopia

**DOI:** 10.1186/1478-4491-7-29

**Published:** 2009-04-01

**Authors:** Marion E McNabb, Cynthia A Hiner, Anne Pfitzer, Yassir Abduljewad, Mesrak Nadew, Petros Faltamo, Jean Anderson

**Affiliations:** 1 Jhpiego, an affiliate of Johns Hopkins University, Baltimore, MD, USA; 2 Jhpiego, an affiliate of Johns Hopkins University, Ethiopia country office, Addis Ababa, Ethiopia; 3 United States Centers for Disease Control and Prevention, Ethiopia country office, Addis Ababa, Ethiopia; 4 Johns Hopkins University School of Medicine, Department of Obstetrics and Gynecology, Baltimore, MD, USA

## Abstract

**Background:**

The Federal Ministry of Health of Ethiopia is implementing an ambitious and rapid scale-up of health care services for the prevention, care and treatment of HIV/AIDS in public facilities. With support from the United States President's Emergency Plan for AIDS Relief, 38 830 service providers were trained, from early 2005 until December 2007, in HIV-related topics. Anecdotal evidence suggested high attrition rates of providers, but reliable quantitative data have been limited.

**Methods:**

With that funding, Jhpiego supports a Training Information Monitoring System, which stores training information for all HIV/AIDS training events supported by the same funding source. Data forms were developed to capture information on providers' working status and were given to eight partners who collected data during routine site visits on individual providers about working status; if not working at the facility, date of and reason for leaving; and source of information.

**Results:**

Data were collected on 1744 providers (59% males) in 53 hospitals and 45 health centres in 10 regional and administrative states. The project found that 32.6% of the providers were no longer at the site, 57.6% are still working on HIV/AIDS services at the same facility where they were trained and 10.4% are at the facility, but not providing HIV/AIDS services. Of the providers not at the facility, the two largest groups were those who had left for further study (27.6%) and those who had gone to another public facility (17.6%). Of all physicians trained, 49.2% had left the facility. Regional and cadre variation was found, for example Gambella had the highest percent of providers no longer at the site (53.7%) while Harari had the highest percentage of providers still working on HIV/AIDS (71.6%).

**Conclusion:**

Overall, the project found that the information in the Training Information Monitoring System can be used to track the working status of trained providers. Data generated from the project are being shared with key stakeholders and used for planning and monitoring the workforce, and partners have agreed to continue collecting data. The attrition rates found in this project imply an increased need to continue to conduct in-service training for HIV/AIDS in the short term. For long-term solutions, retention strategies should be developed and implemented, and opportunities to accelerate the incorporation of HIV/AIDS training in pre-service institutions should be explored. Further study on reasons why providers leave sites and why providers are not working on HIV at the sites where they were trained, in addition to our project findings, can provide valuable data for development of national and regional strategies and retention schemes. Project findings suggest that the development of national and region-specific human resources for health strategy and policies could address important human resources issues found in the project.

## Background

Ethiopia has a population of approximately 77 million people, with nearly 83% living in rural areas [1]. Ethiopia does not have a sufficient health care workforce to meet the population's demand for services and the burden of disease. The country is far below the World Health Organization (WHO) recommended standard of one physician for every 10 000 persons, with a total of 2115 physicians – translating to only one physician for 36 407 persons. In terms of nurses, Ethiopia is above the WHO-recommended one nurse per 5000 people, with one nurse per 4314 individuals [2]. However, a substantial proportion of nurses are junior nurses with only certificate-level (two years) qualification.

The existing human resources (HR) for health crisis can significantly impede Ethiopia's attempts to attain ambitious targets to scale up primary health care services, as well as to reach goals for universal access to HIV/AIDS prevention, care and treatment services by 2010. A 2003 study noted that, given current HR, there would be a 14%–66% gap in physicians necessary to address both basic health services and direct HIV/AIDS care needed to reach HIV/AIDS prevention, care and treatment goals of the Ethiopian government, the United States President's Emergency Plan for AIDS Relief (PEPFAR) and the Global Fund to Fight AIDS, Tuberculosis and Malaria [3].

Human capacity building – including training service providers in all areas of HIV prevention, care and treatment – is a significant component of PEPFAR Ethiopia support to the Federal Ministry of Health (FMOH) HIV/AIDS programme. From April 2007 to March 2008, PEPFAR planned to support training of 124 920 persons in all areas of HIV/AIDS prevention, treatment, care and support (United States Government Country Operational Plan for Ethiopia for PEPFAR 2007). While not all of these individuals are health care workers – many are community-based workers and other cadres of workers – this still represents a massive training and human capacity building effort for HIV/AIDS. Over the past two years, training budgets accounted for 30%–40% of the total PEPFAR funding for Ethiopia.

With funding from PEPFAR, through the United States Centers for Disease Control and Prevention (CDC), Jhpiego supports a Training Information Monitoring System (TIMS^©^), which stores key participant and training information for all PEPFAR-funded training events. This paper describes a project that uses training data from the TIMS database to follow up providers after training to assess whether they are still working in HIV/AIDS-related services. The objective of this project was to assess the usefulness and feasibility of collecting these data for monitoring and planning of HIV/AIDS services.

## Methods

### Implementation of the project

TIMS is a Microsoft Access database designed by Jhpiego in 1997 to track Jhpiego's competence-based training courses, capturing key information on participants, trainers and courses. TIMS in Ethiopia tracks course information by PEPFAR-implementing partners and links courses to an overall focus area that matches PEPFAR focus areas. The United States Agency for International Development (USAID) and CDC Ethiopia require that PEPFAR-implementing partners report training data for entry into TIMS. As of the beginning of the project in January 2007, 26 partners had reported training information – totaling 31 284 trained providers and 1038 courses.

With input from key stakeholders, Jhpiego developed a tool to collect information about trained providers on: HIV/AIDS working status (yes/no); percentage level of effort for HIV/AIDS services; if not working on HIV/AIDS, reasons why not and date of departure from the facility (when applicable); and the method of collecting data (orally, from official HR records, etc.). Each tool was completed for the individual hospital or health centre with information on the service provider trained, date of training, course name, profession and gender. Jhpiego staff pretested this data collection tool prior to the project and made minor changes before the project's rollout.

Eight PEPFAR partners, including Jhpiego, were selected to participate in the project based on their role in the direct implementation of clinical HIV/AIDS services for hospitals and health centres. Since the selected partners regularly visit sites where HIV/AIDS services are delivered, the planned supervision visits were used to collect data for the project. Data collection occurred between February and June 2007. The original data set chosen for the project included 6353 providers who were either trained by the partners participating in the project or were working at the facilities where those partners were supporting HIV service implementation. Public and military hospitals were included in the data set because PEPFAR supports HIV/AIDS services at these facilities. In addition, some of the partners participating in the project had been asked by the MOH's HIV/AIDS Prevention and Control Office and other government stakeholders to support HIV services at private hospitals; thus, the analysis also included trained HIV/AIDS service providers from private hospitals.

Jhpiego provided the partners with forms containing the name, profession and courses attended for each of the participants so that follow-up data could be collected on an individual basis at each facility. In some cases, training information was not yet entered into TIMS, and the partners collected all training information about the providers and their HIV/AIDS working status during their routine site visits. The method of data collection (e.g. administrator's official record, orally from administrator, etc.) was also recorded to help understand the quality of HR records kept by the hospital and to monitor the quality of data collected. The completed forms were sent to Jhpiego, where Jhpiego staff compiled and entered the data and conducted the analysis by means of SPSS and SAS software.

Some partners reported being unable to find information on some providers, and these providers were removed from the analysis. In addition, if partners did not have scheduled visits to some of the facilities they supported, data were not collected from those sites and those providers were excluded from the analysis.

The partners were asked to complete an evaluation form to gather feedback on the usefulness of the data, the ease or difficulty of collecting the data, and the perceived use of these data during regular programme monitoring. This information will be used to help determine whether this type of data collection will occur on a routine basis as part of the TIMS programme.

### Data analysis

The responses for several variables – training focus, qualification and reason for not being at the site – were grouped into "other" if the sample size was small and represented less than 2% of the total sample. Approximately 26% of the responses for reasons why they were not working at the site were "don't know," and these responses were left in the analysis because they represent data where it was known people had left, but the reason was unknown. For people who were working in HIV, data were collected on their percentage of time working in HIV (1%–100%); however, for this analysis everyone was grouped as working in HIV regardless of their levels of effort.

Attrition in this analysis is defined as the provider's no longer working at the site at the time of the supervision visit. Responses for reason for leaving included: left for further study, joined a nongovernmental organization (NGO), joined the private sector, transferred to another public facility, other (which included changed professions, left the country, deceased) and unknown. Breaking down attrition as defined above, there are two groups of health care workers: those leaving the public health sector entirely or for reasons unknown, and those transferring to another public facility. Of those who transferred to another public facility, we are unsure if they are still providing HIV/AIDS services, and there are no formal systems for providers who transfer to a new facility to report prior training courses they have attended. They are considered part of the attrition category. Ultimately, in the overall analysis, we focus on the providers who have left the site (32% of the total trained), since the loss of those providers requires managers to train new providers as replacements.

The data were analysed by means of SAS software. Summary statistics were generated with gender, type of provider, type of facility, working status and reasons for leaving as the categorical variable. Chi-square was performed to analyse comparisons by variables potentially related to attrition. For the chi-square analysis, attrition was further grouped by HIV-working status into "working in HIV" and "not working in HIV." The latter category included individuals who were at the site but were not providing HIV services, grouped with those who were no longer at the site. The chi-square was also performed using a grouping of those "at the site" and "not at the site", but the results of the analysis were similar. The HIV-working status was used because it more accurately reflects the concept being discussed. Working status was analysed based on work location by grouping regions into established regions (Addis Ababa; Amhara; Dire Dawa; Harari; Oromia; Southern Nations, Nationalities and Peoples States (SNNPR)); Tigray; and emerging regions (Afar, Benishengul Gumuz, Gambella and Somali). We also reviewed working status by qualification and by number of courses taken.

## Results

Of the 6353 service providers with information provided, a total of 1744 service providers were followed up from 53 hospitals and 45 health centres in 10 of 11 regional and administrative states. Nearly 88% of the providers worked in a hospital and 59% were male. Fifty-seven percent were nurses and/or midwives, while 14% were physicians; all other professions each represented less than 10% of the sample. Of the 1744 providers, 1344 had taken only one HIV course, while the remaining 400 had taken at least two courses (Table 1). Of the recorded data collection methods, most data were collected orally through conversations with either a health worker (56%), a site administrator (14%) or the trained individual themselves (16%).

**Table 1:** Characteristics of health care workers followed-up, by region

**Region (n)**	**Worked at a hospital**		**Male**		**Physicians or specialists**		**Nurses and/or midwives**		**Took two or more classes**	
Addis Ababa (334)	285	85.6%	162	48.5%	73	21.9%	181	54.2%	81	24.3%

Afar (30)	22	76.7%	20	66.7%	6	20.0%	16	53.3%	10	33.3%

Amhara (138)	138	100%	81	58.7%	25	18.1%	79	57.3%	30	21.7%

Benishangul-Gumuz (84)	84	100%	66	78.6%	12	14.3%	48	57.1%	25	29.8%

Dire Dawa (63)	49	77.8%	35	55.6%	9	14.3%	42	66.7%	8	12.7%

Gambella (41)	41	100%	34	82.9%	4	9.8%	27	65.9%	16	39.0%

Harari (194)	194	100%	108	55.7%	18	9.3%	141	72.7%	12	6.2%

Oromia (227)	84	37.0%	127	56.0%	8	3.5%	161	70.9%	19	8.4%

SNNPR (474)	473	99.8%	291	61.4%	60	12.7%	216	45.6%	144	30.4%

Tigray (159)	159	100%	107	67.3%	25	15.7%	87	54.7%	55	34.6%

**Total (1744)**	**1529**	**87.8%**	**1031**	**59.1%**	**240**	**13.8%**	**998**	**57.2%**	**400**	**22.9%**

Of the providers, 57.6% were found at the site and working with some level of effort on HIV/AIDS services; 10.4% of the providers were working at the site, but reported not to be working on HIV/AIDS services; and 32% were no longer working at the site (Table 2). Of the 32% who had left the site, reasons for leaving included joining the private sector (10.2%), joining an NGO (9.7%), leaving for further study (27.6%), working in another public facility (17.6%), other (8.6%) and unknown (26.3%) (Table 3).

**Table 2:** Working status of health care workers followed-up, by region

**Region (n)**	**No longer working at the site**		**At the site but not working in HIV**		**At the site and working in HIV**	
Addis Ababa (334)	103	30.8%	55	16.5%	176	52.7%

Afar (30)	15	50.0%	2	6.7%	13	43.3%

Amhara (138)	33	23.9%	11	8.0%	94	68.1%

Benishangul-Gumuz (84)	39	46.4%	13	15.5%	32	38.1%

Dire Dawa (63)	12	19.0%	0	0.0%	51	81.0%

Gambella (41)	22	53.7%	5	12.2%	14	34.1%

Harari (194)	43	22.2%	12	6.2%	139	71.6%

Oromia (227)	62	27.3%	20	8.8%	145	63.9%

SNNPR (474)	185	39.0%	33	7.0%	256	54.0%

Tigray (159)	44	27.7%	30	18.9%	85	53.5%

**Total (1744)**	**558**	**32.0%**	**181**	**10.4%**	**1005**	**57.6%**

**Table 3:** Reasons why health care workers are no longer at their sites, by region

**Region**	**Further Study**		**Joined Private Sector**		**Joined NGO**		**Another Public Facility**		**Other**		**Unknown**	
Addis Ababa (103)	20	19.4%	9	8.7%	13	12.6%	12	11.7%	16	15.5%	33	32.0%

Afar (15)	7	46.7%	2	13.3%	5	33.3%	0	0.0%	0	0.0%	1	6.7%

Amhara (33)	10	30.3%	1	3.0%	2	6.1%	6	18.2%	1	3.0%	13	39.4%

Benishangul-Gumuz (39)	10	25.6%	4	10.3%	5	12.8%	10	25.6%	3	7.7%	7	17.9%

Dire Dawa (12)	6	50.0%	2	16.7%	0	0.0%	1	8.3%	1	8.3%	2	16.7%

Gambella (22)	13	59.1%	1	4.5%	3	13.6%	1	4.5%	0	0.0%	4	18.2%

Harari (43)	7	16.3%	2	4.7%	3	7.0%	5	11.6%	9	20.9%	17	39.5%

Oromia (62)	24	38.7%	3	4.8%	6	9.7%	17	27.4%	2	3.2%	10	16.1%

SNNPR (185)	49	26.5%	23	12.4%	10	5.4%	36	19.5%	10	5.4%	57	30.8%

Tigray (44)	8	18.2%	10	22.7%	7	15.9%	10	22.7%	6	13.6%	3	6.8%

**Total (558)**	**154**	**27.6%**	**57**	**10.2%**	**54**	**9.7%**	**98**	**17.6%**	**48**	**8.6%**	**147**	**26.3%**

Of all the providers no longer at the facility at the time of the visit, 82.4% were no longer working in the public sector and can be considered "lost," while 17.3% transferred to another public facility and were still part of the public sector health care workforce. Data were not collected on the 17.3% of the providers, so it is not known if they were working in HIV/AIDS services. For the different chi-square analyses, working status was grouped into "working in HIV" (57.3%) and "not working in HIV" (42.7%). This was analysed by established regions versus emerging regions. We found that providers who had been working at facilities in emerging regions when they were trained were less likely still to be working at those sites than those working in established regions (p < .0001).

In addition to an analysis by region, we explored attrition and the reported destination by profession. Pharmacists and lab personnel were the most likely to have joined the private sector (18% and 19%, respectively); while 32% and 28%, respectively, had transferred to other public facilities. Sixteen percent of physicians joined the private sector, and were also the most likely (19%) to have joined an NGO. Nurses and/or midwives were the most likely to have left for further study (35%). (This is likely correlated with the implementation of the Accelerated Training for Health Officers, a key national strategy to expand the number of this new cadre to provide curative services at health centres and to manage woreda (district) health offices. Diploma-level health care workers are eligible for the accelerated programme.) However, when overall working status by nurses and/or midwives was compared to all other professions, the difference was statistically significant for physicians and specialists (p < .0001) and administrators and management (p = .0091), with both groups less likely still to be working in HIV than those in the nurse and/or midwife group (Table 4). Finally, we found that providers were just as likely to be working if they had attended one training course or more than one training course.

**Table 4:** Work status of health care workers, by profession

**Profession**	**No longer working at the site**		**At site but not working in HIV**		**At site and working in HIV**	
Administrator/management (62)	28	45.2%	7	11.3%	27	43.5%

Physician/specialist (240)	118	49.2%	18	7.5%	104	43.3%

Health officer (79)	19	24.1%	7	8.9%	53	67.1%

Nurse, midwife, nurse/midwife (998)	271	27.2%	125	12.5%	602	60.3%

Pharmacist/druggist (100)	38	38.0%	9	9.0%	53	53.0%

Laboratory (136)	43	31.6%	5	3.7%	88	64.7%

Sanitarian/environmental health (46)	19	41.3%	1	2.2%	26	56.5%

Other or unknown (83)	22	26.5%	9	10.8%	52	62.7%

**Total (1744)**	**558**	**32.0%**	**181**	**10.4%**	**1005**	**57.6%**

Sixty percent of the partners found that collecting these data during site visits was not burdensome. All partners agreed to collect this information in the future and recommended that these data be collected on a semiannual basis. Eighty percent of partners reported that they would use the information for planning and monitoring purposes.

## Discussion

Given the significant resources devoted to training health care workers in Ethiopia, tracking whether providers trained to deliver HIV/AIDS services are indeed providing those services in public facilities is important for monitoring and evaluation of programme implementation and for programme planning. TIMS in Ethiopia provides an opportunity to capture this information in a meaningful and collaborative context.

This project noted a very high attrition rate, with 32% of providers having left their original work sites – overall, 24.6% were no longer in the public sector. This rate is alarmingly high, and further exploration into "push and pull" factors is warranted. Physicians had the highest attrition, with more than 49% no longer at the sites and fewer than 6% having transferred to another public facility. This rate is almost twice as high as that found in a 2006 study from Ethiopia's FMOH, which reported an attrition rate from the public sector of 26% for physicians [4]. Another report calculated an attrition rate of 9.6% among physicians in Ethiopia by comparing the number of physicians graduating in a year and the number working in a year [3].

Ten percent of health providers who had left the facility were reported to have joined the private sector, and an additional 10% were reported to have joined an NGO. The 10.6% of providers who remained at the site but were not working in HIV requires further attention and emphasizes the potential need to better target training resources and improve selection of health care workers to be trained. In one region, Tigray, where 18.9% of workers were reassigned within a facility, staff from the Regional Health Bureau reported that they considered training as a tool to improve retention (Tadesse Y: Report on a Retention Fact Finding Visit to Mekelle. December 2007). This finding suggests that other factors beyond HIV/AIDS-related service delivery needs may influence the selection of providers for training.

We also found considerable regional variation in attrition rates. Recognition of these trends and regional differences can influence decision-making at the regional and national levels to ensure that HIV/AIDS services are adequately staffed.

Through this project, we found that using information gathered in a standardized way by PEPFAR partners during routine supervision visits and entered into the TIMS database demonstrates an innovative and effective way to monitor the HIV/AIDS workforce. Such monitoring contributed to the partners' ability to ensure coverage of trained providers to deliver services and helped inform planning for replacement training, where applicable, at little additional cost. Partners requested region- and site-specific analyses for use in planning for replacement training and have also used these data to negotiate with regional health bureaux for effective retention strategies. The collection of all training data in one central database allowed access to a comprehensive list of providers trained at individual sites, regardless of which organization supported the training, and facilitated comprehensive site-level HIV/AIDS workforce assessments.

In the absence of a national human resource information system (HRIS), the findings of this project provided the potential to look at the working status of a large country-wide cohort of providers and helped facilitate and inform planning by regional health bureaux and the FMOH, which will ensure that adequate numbers of staff are available to deliver comprehensive HIV/AIDS services and develop evidence-based retention strategies.

Furthermore, routine analysis of TIMS follow-up data may inform and provide a means to monitor the effectiveness of retention strategies. The TIMS database can also facilitate mapping of the workforce to support the use of these data for general as well as HIV/AIDS-specific workforce planning.

The attrition found through this project suggests that a stop-gap approach to train more service providers may be necessary to meet HIV/AIDS service demand. However, this is not a sustainable solution and requires significant resources to maintain a standard level of trained providers. Other options include task shifting of certain care responsibilities to lower-level cadres of health care workers or creation of a specialized cadre of HIV/AIDS workers. Various retention schemes should be developed, implemented and evaluated at both a national and regional level. Current human resource practices in the health sector should be reviewed and national guidelines/policies regarding human resources for health should be developed to address problem areas identified through this project.

As the need for HIV/AIDS services continues to increase in Ethiopia, more trained professionals will be needed, highlighting the need to focus efforts towards greater integration of HIV/AIDS content into pre-service education to ensure that new graduates have the base knowledge, skills and attitudes to provide various HIV/AIDS-related services.

### Limitations

Despite the large TIMS data set of trained providers, there is a significant lag time for reporting and data entry into TIMS, with only 38 380 persons in the database as of December 2007, compared to the projected 124 920 people who are to be trained by March 2008. However, the lag time is comparable across partners and regions and, therefore, the sample analysed in this study is believed to be representative.

It is important to note that the numbers generated concerning attrition in this project are specific to providers trained in HIV/AIDS services in Ethiopia, and may not be generalized to other service areas or the Ethiopian workforce in general. There may be some characteristics of these individuals that make them different from other health care workers who have not had HIV/AIDS training opportunities.

The majority of data was collected orally from health care workers rather than from administrative records. Data were collected from official HR records at only a few sites, highlighting the need to support site-level HR management at public facilities. Information was not available for the individuals who had left sites where they had received training, but had transferred to another public sector site. It is possible they are still working in HIV and, therefore, do not represent true attrition from the HIV/AIDS public health care sector. However, their transfer still has significant site-level implications for maintaining services and high-quality care.

Finally, data were not collected on the reasons why providers left the facility, in addition to where they went, or were still at the facility but not working on HIV/AIDS, limiting the scope of information usable to develop targeted retention schemes.

## Conclusion

The project successfully demonstrated that data found in TIMS can be used for following providers trained in HIV/AIDS to assess their HIV/AIDS working status and evaluate attrition. With longer follow-up, there is an opportunity to collect information on time and geographical trends. Key government stakeholders are actively using this information for HIV/AIDS workforce planning.

Based on the project findings, we recommend that tracking of trained HIV/AIDS health care workers through the use of TIMS be rolled out to all health care facilities. The data should be routinely analysed and disseminated to key stakeholders, to inform planning for HIV/AIDS training needs and development of retention schemes. Data on the reasons why providers leave the facility and why trained providers are not working on HIV at the facility post training should be collected to have a broader understanding of the "push and pull" factors in order to develop targeted retention schemes.

The study findings also highlight the need for national and regional specific policies and guidelines regarding human resources for health to be developed and implemented at all levels to address problem areas found in the study. This study points out the need to strengthen and expand pre-service programmes aimed at incorporating HIV/AIDS competences into the curriculum. This study also highlights the need for the FMOH and individual regions to consider a national HR information system to track movements of all health workers in Ethiopia.

## Competing interests

The authors declare that they have no competing interests.

## Authors' contributions

MEM contributed to the conception, design, implementation and analysis of project findings and dissemination of findings, as well as writing the manuscript. CAH contributed to the conception, design and statistical analysis of project data as well as overall technical and monitoring-and-evaluation-related support for the project and writing the manuscript. AP contributed to the conception, design and implementation of the project as well as overall guidance and oversight of project implementation and dissemination of findings. AP also contributed to the manuscript. YA contributed to the conception, design, implementation and overall guidance and support for the project. YA also contributed to and reviewed the manuscript. MN contributed to the implementation and analysis of the findings and reviewed the manuscript. PF contributed to the conception, design, implementation and overall support for the project. PF also reviewed the manuscript. JA contributed to the design and overall support for project implementation. JA also critically reviewed the manuscript and has given the final approval for Jhpiego/JHU publication.
